# Eotaxins and Their Receptor in Colorectal Cancer—A Literature Review

**DOI:** 10.3390/cancers12061383

**Published:** 2020-05-28

**Authors:** Monika Zajkowska, Barbara Mroczko

**Affiliations:** 1 Department of Neurodegeneration Diagnostics, Medical University of Bialystok, 15-269 Bialystok, Poland; mroczko@umb.edu.pl; 2 Department of Biochemical Diagnostics, Medical University of Bialystok, 15-269 Bialystok, Poland

**Keywords:** CCL11, CCL24, CCL26, CCR3, CRC

## Abstract

Colorectal cancer (CRC) is one of the most common malignancies in the world, with a global incidence of almost 2 million new cases every year. Despite the availability of many diagnostic tests, including laboratory tests and molecular diagnostics, an increasing number of new cases is observed. Thus, it is very important to search new markers that would show high diagnostic sensitivity and specificity in the detection of colorectal cancer in early stages of the disease. Eotaxins are proteins that belong to the cytokine group—small molecules with a variety of applications. Their main role is the activation of basophils and eosinophils involved in inflammatory processes. Therefore, we performed an extensive search of the literature pertaining to our investigation via the MEDLINE/PubMed database. On the basis of available literature, we can assume that eotaxins accumulate in cancer cells in the course of CRC. This leads to a decrease in the chemotaxis of eosinophils, which are effector immune cells with anti-tumor activity. This may explain a decrease in their number as a defense mechanism of cancer cells against their destruction and may be useful when attempting anti-tumor therapy with the use of chemokines.

## 1. Introduction

### 1.1. Colorectal Cancer

Colorectal cancer (CRC) is a disease which usually develops as a result of uncontrolled cell growth in a specific part of the large intestine. Presence of a tumor in the majority of patients is asymptomatic, and therefore early diagnosis, which is currently limited to screening methods such as fecal occult blood testing, flexible sigmoidoscopy and colonoscopy, is vital. Tumor markers such as carcinoembryonic antigen (CEA) or carbohydrate antigen 19-9 (CA 19-9) are also used in CRC diagnostics, but their diagnostic sensitivity and specificity are insufficient. Therefore, many scientists focus their efforts on the search for new markers that could facilitate CRC detection in the future and significantly impact the lifespan and quality of life of patients [1,2,3].

#### 1.1.1. Epidemiology

According to the World Health Organization (WHO), the global incidence of colorectal cancer (CRC) is almost 2 million new cases per year, with approximately 880,000 deaths annually. WHO predicts that in 2040, the number of new CRC cases will exceed 3 million, with the number of fatalities reaching 1.5 million per year. Despite the fact that CRC ranks second in terms of incidence rates among men and third among women, incidence expressed as a percentage is higher for men and accounts for almost 11%, while for women it is approximately 9.5%. Colorectal cancer is more common in developed than in developing countries. In developing countries which are witnessing economic advancement, adoption of a ’Western lifestyle’ and dietary habits characterized by a higher intake of red meat, fat and total calories, along with increasing life expectancy and population growth, heralds a significant increase in CRC burden. A rise in CRC incidence in developing countries is also attributed to environmental changes prompted by economic transition [4,5,6,7,8].

#### 1.1.2. Pathogenesis

The seminal and classical tumor progression model of Fearon and Vogelstein based on a polyp to cancer progression sequence was the first attempt to describe molecular mechanisms of colorectal carcinogenesis. This model involves a few steps of cancer progression. The first is the formation of benign neoplasms, followed by progression of benign neoplasms to malignant neoplasms and, finally, transformation to invasive carcinoma. Introduction of this model significantly facilitated the understanding of molecular tumorigenesis mechanisms associated with accumulation of genetic and epigenetic changes that transform normal glandular epithelial cells into invasive adenocarcinomas. The basis for the development of colorectal cancer with corresponding accumulation of genetic changes is the adenoma-carcinoma sequence. It is caused by three major pathways: CIN (chromosomal instability), MSI (microsatellite instability) and CIMP (CpG island methylator phenotype) [9].

The CIN pathway is observed in around 85% of adenoma-carcinoma transitions and appears to be the most common type of genetic instability in CRC. It leads to the presence of aneuploidy or polyploidy, by gains or losses of whole or large portions of chromosomes. CIN tumors can be recognized by the accumulation of mutations in specific oncogenes, i.e., KRAS protooncogene GTPase (KRAS) and B-Raf protooncogene serine/threonine kinase (BRAF), and tumor suppressor genes, such as adenomatous polyposis coli (APC) and tumor protein p53 (TP53) [9,10,11].

The MSI pathway is characterized mainly by mutations or epigenetic changes of genes that maintain genetic stability. Microsatellites are repetitive DNA sequences consisting of tandem repeats (usually 1–5 base pairs). Patients with the MSI phenotype exhibit a high frequency of replication errors, particularly in repetitive DNA sequences, primarily due to the slippage of the DNA polymerase. Approximately 15% of sporadic CRCs are thought to be caused by this mechanism. CIN tumors can be recognized by the accumulation of mutations in tumor-suppressor genes (i.e., transforming growth factor beta receptor 2 (TGFRII), Insulin-like growth factor 2 receptor (IGF2R) and Bcl-2-associated X protein (BAX)). Additionally, the MSI pathway has its own recommendation in the Bethesda Guidelines. To access the MSI status of cancer, a standard panel of five microsatellite markers (BAT26, BAT25, D2S123, D5S346 and D17S250) repeats should be present [9,10,11,12,13].

In the CIMP pathway, epigenetic instability is manifested as global DNA hypermethylation and hypermethylation of loci that contain CpG islands. Low methylation is present in all CRC cases, but approximately 10–35% of patients are characterized by an exceptionally high proportion of aberrantly methylated CpG loci. CIMP mechanisms are still unknown, although many researchers have been exploring them, with some demonstrating a correlation between the overexpression of DNA methyltransferases DNMT3B or DNMT1 and CIMP. Interestingly, CIMP and MSI pathways often occur simultaneously [9,11,13].

Many patients diagnosed with colorectal cancer do not notice any symptoms of the disease. One of the most common symptoms is the presence of blood in or on the stool and the associated iron deficiency anemia (fatigue, weakness). Other symptoms depend on the primary tumor location [14,15].

#### 1.1.3. Classification

Currently, three types of classifications are used for CRC—WHO, Tumor Node Metastasis (TNM) and molecular. Other classification systems can also be distinguished including histological classification and grading (G1–4), and two historical staging systems not currently used (Duke’s and Astler-Coller’s classifications) [16].

The WHO classification (4th edition) introduced a new histological division of cancer into epithelial, mesenchymal, secondary tumors and lyphomas. Epithelial tumors can be divided into premalignant, serrated lesions, hamartomas, carcinomas and neuroendocrine neoplasms. The most important subgroup are carcinomas. This subgroup can be divided into adenocarcinomas (around 90% of all cases), adenosquamous, spindle cell, squamous cell and undifferentiated carcinomas [17].

The pathological TNM staging of colorectal cancer is based on the eighth edition of the American Joint Committee on Cancer (AJCC) staging manual. This most popular system for assessing the stage and spread of cancer combines information regarding not only the tumor itself or nearby lymph nodes, but also distant metastases. Category T refers to the size and location of the neoplastic lesion and spread to nearby tissues, category N determines regional lymph node involvement of the tumor, while category M identifies the presence of metastases to distant tissues and organs [16,18,19].

The consensus molecular classification (CMS) divides CRC cases into four subtypes (CMS1–4), on the basis of comprehensive gene expression profiles. This new classification system integrates six classifications based on comprehensive gene expression levels of stage I–IV CRCs. Each of the four subtypes has a characteristic molecular background, which has been demonstrated to be a prognostic factor. More detailed information on CMS subtypes is presented in Table 1 [20,21,22].

One of the basic components of the pathological description of a tumor with prognostic significance is the degree of histological malignancy. There are five degrees: G1 (most differentiated cancer, better patient prognosis), G2 (medium differentiated), G3 (poorly differentiated, worse patient prognosis), G4 (undifferentiated) and Gx (grade cannot be determined) [16].

#### 1.1.4. Screening and Diagnostics

A lower risk of developing colorectal cancer is associated with a diet rich in vegetables, fruit and whole grain cereal products, as well as physical activity. Environmental factors such as dietary habits, obesity, smoking and heavy alcohol consumption have been found to increase CRC risk [23,24,25]. Several studies have indicated that calcium or vitamin D3 supplementation may have an anti-disease effect [26,27]. In addition, it has been proven that chronic use of nonsteroidal anti-inflammatory drugs (acetylsalicylic acid) reduces the risk of cancer [28]. Not smoking or smoking cessation reduces the risk of developing many cancers, not only colorectal cancer [29].

Screening tests are the standard methods for detecting benign lesions—adenomas (primary prevention) and cancers at an early stage (secondary prevention). There are a few modalities available, i.e., FOBT (faecal occult blood test), FIT (faecal immunochemical test), colonoscopy, sigmoidoscopy, computed tomographic (CT) colonography or multi-target stool deoxyribonucleic acid (mt-sDNA) test [23].

FOBT and FIT, which are the most commonly used tests because of their high availability and non-invasiveness, should be performed every 12 months. However, the tests have some limitations—they do not detect pre-cancerous changes and their sensitivity is fairly low. A positive result is an indication for a colonoscopy. Other screening tests are sigmoidoscopy and CT colonography. These tests are semi-invasive and should be performed every five years (may be combined with FOBT). Both have high sensitivity, but their limitations include unpleasant bowel preparation and higher costs. The highest sensitivity can be obtained using colonoscopy, which should be performed every 10 years. It is the most commonly utilised modality which, however, carries a risk of bowel perforation or bleeding. The multitarget stool DNA (mt-sDNA) test is not commonly used, although it is highly sensitive (comparable to colonoscopy, sigmoidoscopy and CT colonography) and non-invasive, which makes it a very good alternative to invasive tests [23,30].

If colorectal cancer is suspected, the diagnostic process should commence with a thorough physical examination, including a rectal examination. This should be followed by endoscopic procedures which would allow for assessment of the cancer process. Depending on tumor location, a rectoscopy, a sigmoidoscopy or a colonoscopy enable detection of the tumor and possible coexisting changes, as well as allowing for sample collection for histopathological examination. In order to verify the presence of synchronous tumors, every patient with the diagnosis of colorectal cancer should have a complete preoperative colonoscopy. If the examination cannot be performed (i.e., tumor narrowing the intestinal lumen), it should be conducted after surgery [23,31,32,33].

In order to determine the stage of cancer development (surgery, presence of regional lymph node metastases or distant metastases), imaging tests are performed. An ultrasound or CT scan of the abdomen and pelvis, as well as a chest x-ray in the anteroposterior and lateral projection, may constitute the basis for diagnosis. In the case of potentially operable colorectal cancer, a CT scan of the abdomen and pelvis should be accompanied by a CT scan of the chest. In order to accurately determine the severity of rectal cancer (depth of mesorectal infiltration, presence of regional lymph node metastases) and to plan optimal therapeutic management (primary surgery or preoperative radiotherapy), pelvic magnetic resonance imaging is necessary. A transrectal ultrasound scan is not a routinely used modality in preoperative diagnosis of rectal cancer, due to insufficient imaging coverage, and is considered inferior to magnetic resonance imaging. Positron emission tomography (PET-TK) is performed when CT scan results are inconclusive (suspected metastases), or when potentially resectable metastases are present, in order to exclude other metastatic foci [23,31,32,33].

Laboratory tests can also be used in CRC diagnosis. Markers such as CEA and CA 19-9 are routinely determined in patients with colorectal cancer. However, they are not used in screening tests, due to their relatively low sensitivity and diagnostic specificity. Therefore, establishing new markers that would have high diagnostic sensitivity and specificity to detect colorectal cancer at its earliest stage is important [34].

### 1.2. Chemokines and Their Receptors

Chemokines belong to a group of secreted proteins with the common name of cytokines. The term ’chemokines’ was introduced in 1992 during the Third Symposium of Chemotactic Cytokines and comes from the words ’chemoattractant cytokines’, which refer to their originally described chemoattractant function. They are a large family of approximately 50 members, which have low molecular mass (8–12 kDa) and are composed of 66–111 amino acids [35,36,37]. Their main role is to direct the recruitment and migration of cells to sites of inflammation or injury. They are divided into four classes (XC, CC, CXC, CX3C), according to the placement and number of cysteine residues at the amino terminus. All chemokines have at least two cysteines and almost all (except XCL1 and XCL2) have at least four cysteines [36,38].

The CC chemokine group is further divided into two subgroups—MIP (macrophage inflammatory proteins) and MCP (monocyte chemotactic proteins). Both MIP and MCP chemokines are typical chemoattractants for indicated groups, with the ability to interact with monocytes, T lymphocytes, NK cells, eosinophils, basophil, and dendritic cells. Their interaction with neutrophils has not yet been established [39,40]. In the case of the CXC chemokine group, additional division into two subgroups has been introduced, depending on the presence of a Glu-Leu-Arg (ELR) motif at the N-terminus of the molecule. Its presence determines the chemotactic effect on respective effector cells. ELR+ chemokines affect neutrophils and stimulate angiogenesis, while ELR- chemokines act chemotactically on lymphocytes, monocytes, basophils and eosinophils, and inhibit the process of angiogenesis. Among the XC class chemokines, only two proteins are currently known—XCL1 and XCL2. Both are called lymphotactins (alpha and beta) and act as chemoattractants for T lymphocytes. The CX3C class contains only one chemokine—CX3CL1, commonly known as fraktalkine, which affects the migration of NK cells, monocytes, macrophages, CD8+ and CD4+ lymphocytes [35,37,38].

All of these proteins exert their biological effects by interacting with G-protein-coupled transmembrane chemokine receptors which occur on the cell membrane of target cells (specific effector cells). The nomenclature of chemokines and chemokine receptors is derived directly from their classification. At present, there are 19 receptors corresponding to specific groups of chemokines. Despite their large number, these receptors have a very similar structure and are activated in a similar manner to chemokines themselves. They form seven loops penetrating the cell membrane, and the fragments of the second and third loop located on the inner surface of the membrane are bound to the G protein. Relationships between individual receptors are manifested by conserved motifs found in transmembrane domains. All chemokine receptors have two conserved cysteines, one in the N-terminal domain and the other in the third extracellular loop. These cysteines are connected by a sulfide bridge. This binding is necessary for the conformation of the ligand binding pocket [41]. Chemokine receptors can be functionally divided into two groups. The first group is G-protein coupled receptors (GPCR), located on the inner surface of the cell membrane. Fragments of these receptors bind to the G protein which, under the influence of an extracellular ligand, activates intracellular signal transduction systems. There are four subclasses of GPCR receptors—XCR, CCR, CXCR and CX3CR, which bind to corresponding chemokine groups [42,43]. The second group of receptors are atypical chemokine receptors (ACKR), which are mainly expressed by erythrocytes, lymphatic or vascular endothelial cells. This group includes four receptors: ACKR1 (DARC), ACKR2 (CCBP2), ACKR3 (RDC-1) and ACKR4 (CCX-CKR). They are referred to as ‘silent’ receptors, as they do not alter intracellular calcium and do not participate in signal transduction with the G protein, despite structural similarity to classical receptors. They act as chemokine ’scavengers’, quenching their activity and reducing inflammation [44,45].

Chemokine and receptor complexes demonstrate widely varying differences in terms of selectivity and binding. Some chemokines can bind and activate more than one chemokine receptor, and some chemokine receptors can be activated by more than one chemokine ligand, but only between corresponding groups. Therefore, blocking the activity of one chemokine may not be effective, since its receptor may be activated by another chemokine. This mechanism may be important when attempting anti-chemokine therapy [37,46,47].

Despite such a broad division, we can distinguish additional proteins in many subgroups. The MCP subgroup, belonging to the CC chemokine group, additionally includes chemokines called eotaxins (-1, -2, -3), which, like all MCP chemokines, have chemotactic properties [48].

## 2. Results and Discussion

### 2.1. Eotaxins and Their Receptors in Colorectal Cancer

The first eotaxin was discovered in 1994 by Williams et al. [49], at the National Heart and Lung Institute in London. The authors described a new protein which was able to selectively recruit eosinophils [50]. Other researchers confirmed the role of the newly described protein as a potent eosinophil chemoattractant cytokine. They also succeeded in describing its main receptor—CCR3 (CC chemokine receptor 3) [43,51,52]. A few years later, when other eotaxins were described, they were named using Arabic numbers (Eotaxin-1, -2, -3) [53]. Additional information regarding eotaxins is presented in Table 2.

**Figure 1:**
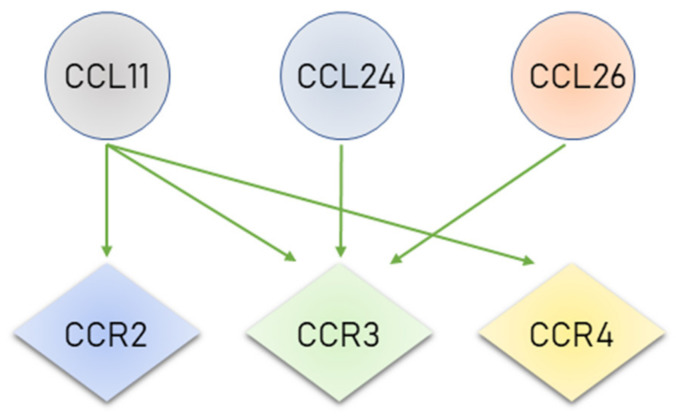
Eotaxins and their receptors.

Eotaxin-2 and -3 can only bind to the CCR3 receptor, but Eotaxin-1 can bind to some other receptors such as CCR2 and CCR4, but it shows highest selectivity for CCR3 (Figure 1) [54].

It has been proven that eotaxins are potent stimulators of some types of cells. Eotaxin-1 (also called CCL 11) is considered a chemoattractant for eosinophils, but not mononuclear cells. The specific eosinophils activated by Eotaxin-1 are mainly implicated in inflammatory diseases, such as atopic dermatitis, allergic rhinitis, asthma and parasitic infections. Eotaxin-2 (CCL 24) is considered a chemoattractant for resting and activated T cells, while Eotaxin-3 (CCL26) is a chemoattractant for eosinophils and basophils and may contribute to the accumulation of eosinophils in atopic diseases [35,55]. Allergic diseases, in which all eotaxins are involved, belong to a group of inflammatory diseases. Therefore, it can be presumed that in all inflammatory diseases in which mainly eosinophils, but also basophils or T lymphocytes are activated, the concentration of these proteins is elevated. Malignant tumors, including colorectal cancer, are one of these diseases. Although the association between eosinophils and cancer was described over a century ago, their exact role in the disease has not yet been defined. Recent observations have revealed that they exhibit regulatory functions towards other immune cells in the tumor microenvironment or direct cytotoxic functions against cancer cells, leading to anti- or pro-tumor activity [56].

**Figure 2:**
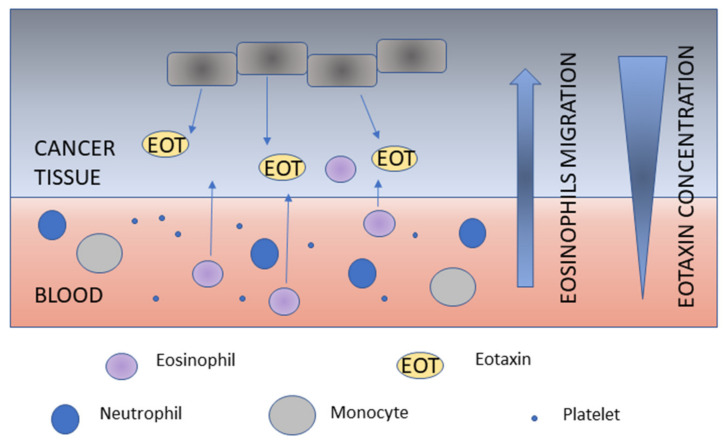
Eotaxins and eosinophils migration.

**Figure 3:**
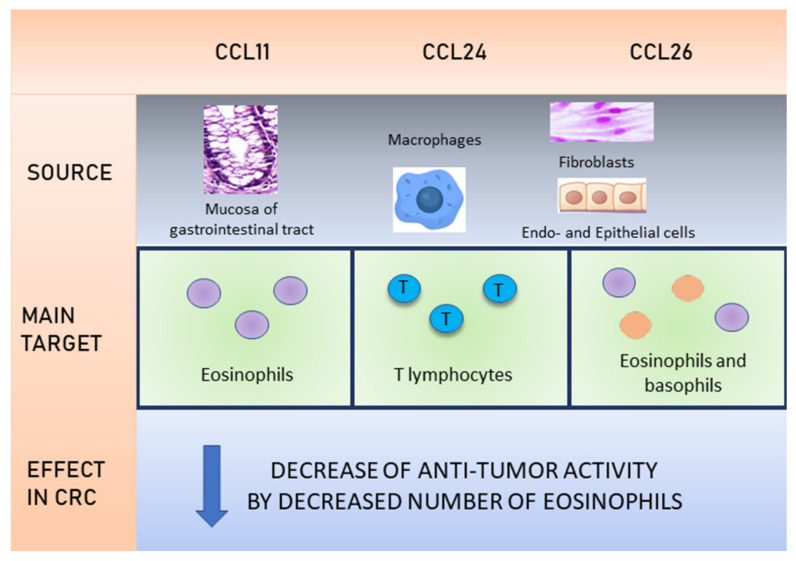
Eotaxins and their role in colorectal cancer.

It can also be presumed that the pathogenetic mechanism of eotaxin participation in CRC development is closely related to the presence of a large number of eosinophils (Figure 2 and Figure 3). These cells are present in tissues with substantial cellular turnover and regenerative capacity, such as colon and rectum, and their presence is critically regulated by eotaxins. This can also explain the presence of eosinophils at sites of wound repair and the commonality of eosinophil infiltrate among solid tumors [57].

A number of researchers have demonstrated that tumor-associated tissue eosinophilia (TATE) or the degranulation of eosinophils is connected with improved prognosis in many types of tumors [58], such as colorectal cancer [59], oesophageal and oral squamous cell carcinomas [60,61], bladder cancer [62] or prostate cancer [63]. Interestingly, some studies on the recruitment of eosinophils in tumors have shown that tissue infiltration by eosinophils can be mediated by factors that can be released from necrotic tumor cells [64,65,66] and some of them may be eotaxins. There are only a few studies which have attempted to investigate the significance of eotaxins in colorectal cancer (CRC).

#### 2.1.1. Eotaxin-1

Physiologically, Eotaxin-1 is expressed in the mucosa of the gastrointestinal tract and may play a role in ulcerative colitis and other gastrointestinal disorders [67]. Importantly, high plasma or serum levels of Eotaxin-1 have been demonstrated in inflammatory bowel disease [68] and colorectal cancer [69,70]. By contrast, Wågsäter et al. [67] found lower concentrations of Eotaxin-1 in 67 CRC patients when compared to 103 healthy subjects. Moreover, in the same paper, the authors reported higher Eotaxin-1 concentrations in tissue lysates from CRC patients in comparison to non-cancerous tissue. To determine its origin, the authors performed immunohistochemistry staining and found immunoreactivity in stromal cells (fibroblasts and leukocytes) [67]. This may indicate that Eotaxin-1 accumulates in tumor tissue. However, due to the divergence in the results obtained by different authors, the findings need to be corroborated in a larger cohort.

Cho et al. [71] found higher expression of Eotaxin-1 in stromal cells, when compared to glandular cells. They indicated that it might help to explain the decreased number of tissue eosinophils, which was also examined in the study, in CRC progression. The authors pointed out that eosinophils are effector immune cells with anti-tumor activity. This may explain a decrease in their number as a defense mechanism against cancer cell destruction. This fact may be useful when attempting anti-tumor therapy with the use of chemokines [71]. Similar results were obtained by Lang et al. [72] The researchers also checked the influence of MS-444 treatment (inhibitor of human RNA-binding (HuR) protein involved in cancer progression) on Eotaxin-1 concentration, but the results were not significant. Different authors have revealed that the in vivo transfer of CD40L into cancer cells induces the expression of some cytokines, including Eotaxin-1. This procedure helps to enhance the anti-cancer effect and increase immunity. This impacts tumor regression, not only locally, but also in remote locations, and contributes to reducing the possibility of tumor metastasis [73].

Interestingly, Krzystek-Korpacka et al. [74] examined differences in the levels of several chemokines, including Eotaxin-1, in the early postoperative period after open and robotic colorectal surgery. They proved that Eotaxin-1 concentrations decreased linearly in the whole cancer group after both types of surgery. Interestingly, after study participants were divided into groups according to the American Society of Anesthesiologists (ASA) physical status classification system, some of them (ASA 1) showed an increase in the concentration of this chemokine after 24 and 72 h. The cause of the increase in Eotaxin-1 concentration in patients without comorbidities (according to the ASA classification system) was not established. However, it can be hypothesized that a healthy organism, as in the case of patients classified as ASA 1, responds with an increase in certain factors faster than an organism burdened with comorbidities. These factors also include chemokines as proteins associated with inflammation resulting from surgery, regardless of the type of surgical procedure. In this regard, it would be interesting to investigate how eotaxin levels present in patients with ASA > 1. In addition, an increase in Eotaxin-1 concentration correlated positively with an increase in IL-1β, TNFα and IL-6 concentrations, and negatively with surgery duration in the case of open colorectal surgery. In regard to robotic surgery, Eotaxin-1 correlated only with IL-1β and TNFα [74].

Studies conducted by Shiels et al. [29] on a mixed group of 1819 prostate, lung, colorectal and ovarian cancer patients demonstrated that cigarette smoking can also affect Eotaxin-1 concentration. Smoking increases it significantly. CCL11 levels were found to be far lower in former smokers, suggesting a decrease in Eotaxin-1 concentration after smoking cessation. Interestingly, in these studies, the number of cigarettes smoked per day and smoking duration were not found to be statistically significant. This is due to the appearance of large numbers of irritants in the lungs and the secretion of CCL11 by eosinophils accumulating in the respiratory tract, leading simultaneously to the generalized inflammatory state of studied patients.

Additionally, studies conducted by Zhu et al. [75] on prostate cancer cell lines (DU-145) revealed that CCL11 can promote cancer cell migration and invasion by the activation of the CCR3-ERK pathway and the upregulation of matrix metalloproteinase 3 (MMP-3). The authors also indicated that knockdown of CCR3 may have an inhibitory effect on the invasion and migration of DU-145 cells. This attenuates the activation of ERK1/2 and expression of metalloproteinase induced by CCL11. Inactivation of the ERK pathway also suppresses the invasion and migration promoted by CCL11, and contributes to decreased MMP-3 expression. The above findings may have important clinical applications as therapies that could block CCL11, and CCR3 may be useful in cancer treatment [75].

Tripathi et al. [76] demonstrated on breast cancer cells a very important role of tissue macrophages (TAMs) in the process of tumorigenesis. During this process, TAMs undergo phenotype, switching to acquire a pro-tumor phenotype and promote tumor progression. They preferentially accumulate in hypoxic/necrotic regions of the tumor and their presence in high numbers is strongly associated with poor patient prognosis. Hypoxic tumor cells exhibit upregulated intracellular levels of eotaxin and oncostatin M, which in turn is accompanied by their enhanced release in the culture supernatant. Interestingly, protein synthesis inhibitor cycloheximide can suppress the release of oncostatin M and eotaxin. This demonstrates that the release of these cytokines is essentially dependent on their de novo synthesis. A blockade of eotaxin/oncostatin M prevents hypoxic cancer cells from recruiting macrophages [76].

#### 2.1.2. Eotaxin-2

The effects exerted by CCL24 on basophils and eosinophils are similar to those produced by CCL11. The main source of Eotaxin-2 in the human body are fibroblasts, epithelial cells and macrophages [77]. Eotaxin-2, the same as Eotaxin-1, shows higher expression in stromal cells in comparison to glandular cells in colorectal cancer tissues [71]. Cho et al. [71] revealed that both of these proteins might help to explain the decreased number of eosinophils in CRC development, since a reduction in their number may constitute a defense mechanism against the destruction of cancer cells. Additionally, Cheadle et al. [78] revealed that Eotaxin-2 is one of the chemokines whose elevated levels were found in biopsy samples of primary colorectal cancer and adjacent liver metastases (as a metastatic tumor of colorectal origin). Interestingly, the surrounding non-neoplastic tissues expressed far less Eotaxin-2, suggesting that the presence of this chemokine may be specific to this particular tumor type and might play a role in the conditioning of the tumor microenvironment. The study also confirmed the reports of other researchers that CCL24 shows high expression in CRC tissues. Some authors have also found that high plasma levels of Eotaxin-2 are exclusively associated with cancer-specific mortality [79].

#### 2.1.3. Eotaxin-3

Eotaxin-3 has similar localization and functions to Eotaxin-2. Physiologically, it is expressed in heart and ovarian tissue, dermal fibroblasts and endothelial cells [77]. In a paper by Lan et al. [80] on CCL26, the authors revealed that Eotaxin-3 has similar properties to those of Eotaxin-2 described by Cheadle et al. [78] Eotaxin-3 showed high expression in colorectal cancer and liver metastatic tissue samples. Additionally, the expression of this protein increased with the TNM stage of cancer and showed a positive correlation with PRL-3 (phosphatase of regenerating liver-3), which is an important factor in CRC invasion and metastasis. Importantly, these parameters were strongly correlated with lymph node metastasis, distant metastasis, poorly differentiated tumor and high TNM stage, which leads to poor prognosis for CRC patients [80]. Both publications may indicate the significance of eotaxins in the course of CRC. Perhaps extensive research, not limited to the expression of the examined proteins in tissues, but also exploring their serum concentrations, conducted on a larger cohort would allow for determination of a cut-off point for eotaxins concentrations, which would significantly improve cancer detection. It is commonly known that CRC is frequently asymptomatic and routine screening could increase the detection rate of this type of cancer and reduce its mortality.

#### 2.1.4. Receptor for Eotaxins

**Figure 4:**
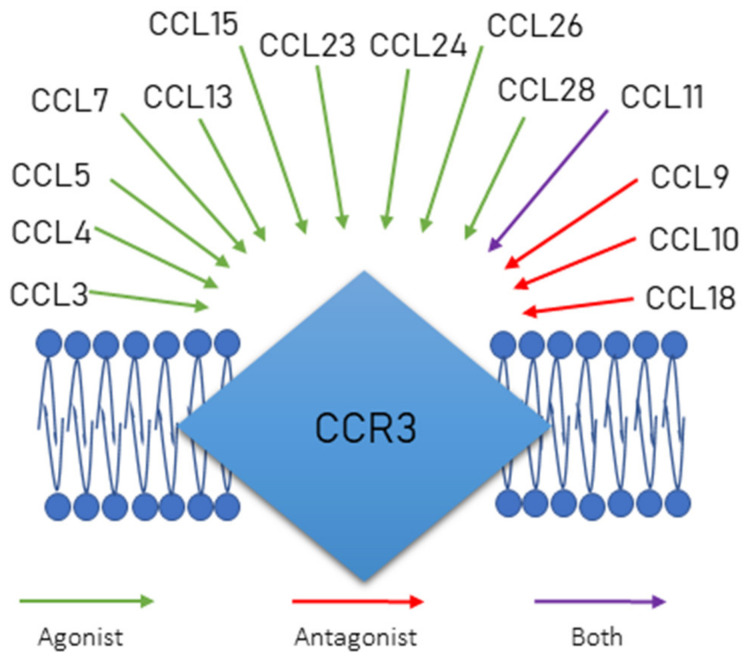
CCR3 and its ligands.

CCR3 is a receptor, not only for eotaxins, but also other chemokines. CCR3 can bind CCL 3, 4, 5, 7, 11, 13, 15, 23, 24, 26, 28 and then it can act as their agonist and, on the other hand, it can also bind to CCL 9, 10, 11, 18 and act as an antagonist for these chemokines (Figure 4). CCR3 can be found on the surface of eosinophils and basophils in blood and on macrophages in the spleen [81].

In a paper by Lan et al. [80], CCR3, similarly to CCL26, showed high expression in tumor tissues (both primary CRC and liver metastases) and was strongly correlated with lymph node metastasis, distant metastasis, poorly differentiated tumor and high TNM stage, which indicates poor prognosis for CRC patients. Cheadle et al. [78] also confirmed CCR3 expression on T lymphocyte cells, which suggest that the immune cells gene can be modified to express a chemokine receptor which has improved tumor-homing abilities. In addition, Cho et al. [82] found that CCR3 expression was significantly higher in liver metastases when compared to their corresponding primary colorectal cancer tissues. The authors suggested that the malignant status of CRC cells might be correlated with CCR3 expression. Their findings were confirmed with the use of HT29 CRC cell line in a paper by Devaud et al. [83], where CCR3 showed high expression. The authors also demonstrated that CCR3 can have an anti-tumor effect correlated with delayed tumor growth. Their research revealed that the pre-incubation of HT29 cells with anti-CCR3 results in a loss of their ability to delay tumor growth.

Interestingly, scientists from Wageningen [84] found that there were differences between male and female mice gene expression in the intestine. One of those genes was the CCR3 gene, which showed dominant expression in female mice. These findings should be taken into consideration in further studies on human tissue, to better understand the mechanism of cancer development in both sexes. These differences may also explain discrepancies in cancer incidence rates between males and females. These may be due to the presence of CCR3, which is a receptor for eotaxins with anti-cancer properties.

In addition, CCL7 is the most commonly described chemokine in combination with CCR3 in the course of CRC. Lee et al. [85] demonstrated that HCT116 and HT29 cell lines show a high expression of CCR3 in patients with CRC and that CCR3 expression is stimulated by high CCL7 expression, particularly in HT29 cells. Other researchers have also indicated an important role of the complex between VEGF-A and CCL7-CCR3 axis as a key node in the extracellular matrix of CRC cells in early metastatic stages. They have demonstrated that chemotaxis of inflammatory cells during this period (from stage II to III TNM) decreases in extracellular matrix and it might be connected with the established connection between CCL7-CCR3 and metalloproteinases (MMPs)/chemotactic factor family [86].

## 3. Literature Search and Data Extraction

**Figure 5:**
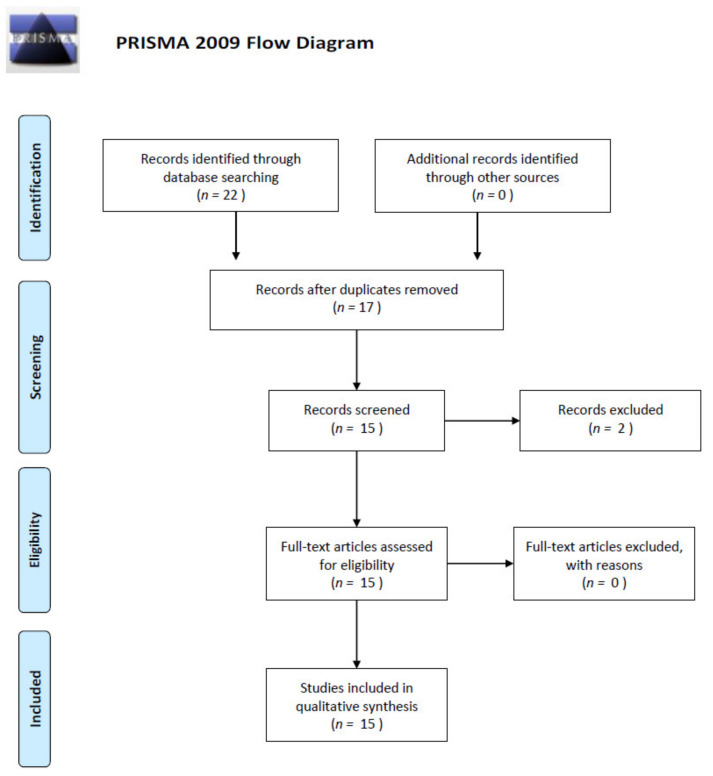
Schematic illustration of articles included in the review.

We performed a comprehensive literature search using the MEDLINE/PubMed electronic database on 24 February 2020, with the following search strategy: “CCL11/CCL24/CCL26/CCR3 AND colorectal cancer”. For CCL11 (Eotaxin-1), we found eight papers. In the case of CCL 24 (Eotaxin-2), we found four papers. For CCL26 (Eotaxin-3), we found only one paper, and for CCR3—nine papers. In the next step, we excluded all papers that were duplicated and non-significant (two papers did not concern CRC and eotaxins). Finally, 15 publications were included in the study. All steps are included in the PRISMA 2009 Flow Diagram (Figure 5) [87].

## 4. Conclusions

Explaining the role of eotaxins in CRC is difficult, due to a very small number of publications on the subject. The majority of published papers indicate that eotaxins and their receptor (CCR3) show high expression in cancer tissues when compared to healthy controls. Serum or plasma concentrations of these parameters show no significant differences between CRC and controls. Therefore, it can be hypothesized that eotaxins accumulate in cancer cells in the course of CRC, leading to a decrease in chemotaxis of eosinophils, which are effector immune cells with anti-tumor activity. This may explain a decrease in their number as a defense mechanism against the destruction of cancer cells. Thus, it is important to continue research on eotaxins and their receptor, in order to confirm these hypotheses.

## Figures and Tables

**Table 1:** Consensus Molecular Classification (CMS) of colorectal cancer.

CMS1—Immune	CMS2—Canonical	CMS3—Metabolic	CMS4—Mesenchymal
• hypermutation and microsatellite instability BRAF mutations immune cell (Th1 lymphocyte cytotoxic T cell, NK cell) infiltration upregulated immune checkpoints (i.e., PD-1)	• high somatic copy number alteration WNT and MYC activation	• dysregulation of metabolic pathways KRAS mutation loss of TH17 cells low somatic copy number alteration	• upregulation of EMT pathways elevated TGF-β signaling matrix remodeling angiogenesis complement activation integrin-β3 upregulation stromal infiltration immune upregulation platelet signatures

**Table 2:** Comparison of Eotaxins.

Characteristics	Eotaxin-1	Eotaxin-2		Eotaxin-3
**Gene localization**	Chromosome 17	Chromosome 7		Chromosome 7
**Binding receptors**	CCR2, CCR3, CCR4	CCR3	CCR3	
**Chemoattraction**	eosinophils	T lymphocytes		eosinophils and basophils
**No of amino acids**	74	78		94
**Mass**	8.4 kDa	8.5 kDa		8.5 kDa
